# Dose escalation in advanced floor of the mouth cancer: a pilot study using a combination of IMRT and stereotactic boost

**DOI:** 10.1186/s13014-021-01842-1

**Published:** 2021-06-29

**Authors:** Tomáš Blažek, Zuzana Zděblová Čermáková, Lukáš Knybel, Pavel Hurník, Jan Štembírek, Kamila Resová, Tereza Paračková, Martin Formánek, Jakub Cvek, Renata Soumarová

**Affiliations:** 1 Department of Oncology, University Hospital Ostrava, Ostrava, Czech Republic; 2 Faculty of Medicine, University of Ostrava, Ostrava, Czech Republic; 3 Department of Pathology, University Hospital Ostrava, Ostrava, Czech Republic; 4 Department of Oral and Maxillofacial Surgery, University Hospital Ostrava, Ostrava, Czech Republic; 5 Department of Otorhinolaryngology, University Hospital Ostrava, Ostrava, Czech Republic; 6 3Rd Faculty of Medicine, Charles University Prague, Prague, Czech Republic; 7 Department of Oncology, University Hospital Královské Vinohrady Prague, Prague, Czech Republic; 8 Institute of Animal Physiology and Genetics, Czech Academy of Sciences, Brno, Czech Republic; 9 Faculty of Medicine, Palacký University Olomouc, Olomouc, Czech Republic

**Keywords:** Oral cavity tumor, Floor of the mouth tumor, CyberKnife, CyberKnife boost, Stereotactic radiotherapy, Hypofractionated boost

## Abstract

**Purpose:**

We evaluated the efficiency and toxicity of stereotactic hypofractionated boost in combination with conventionally fractionated radiotherapy in the treatment of advanced floor of the mouth cancer.

**Methods:**

Thirty-seven patients with advanced stage of the floor of the mouth cancer, histologically confirmed squamous cell carcinoma (p16 negative) ineligible for surgical treatment, were indicated for radiochemotherapy or hyperfractionated accelerated radiotherapy (HART). The radiotherapy protocol combined external beam radiotherapy (EBRT) and a stereotactic hypofractionated boost to the primary tumor. The dose delivered from EBRT was 70–72.5 Gy in 35/50 fractions. The hypofractionated boost followed with 10 Gy in two fractions. For the variables—tumor volume, stage and grade a multivariate analysis was performed to find the relationship between overall survival, local progression and metastasis. Toxicity was evaluated according to CTCAE scale version 4.

**Results:**

After a median follow-up of 16 months, 23 patients (62%) achieved complete remission. The median time to local progression and metastasis was 7 months. Local control (LC) at 2 and 5-years was 70% and 62%, respectively. Progression-free survival (PFS) and overall survival (OS) were 57% and 49% at 2 years and 41% and 27% at 5 years, respectively. Statistical analysis revealed that larger tumors had worse overall survival and a greater chance of metastasis. Log-Rank GTV > 44 ccm (HR = 1.96; [95% CI (0.87; 4.38)]; *p* = 0.11). No boost-related severe acute toxicity was observed. Late osteonecrosis was observed in 3 patients (8%).

**Conclusion:**

The combination of EBRT and stereotactic hypofractionated boost is safe and seems to be an effective option for dose escalation in patients with advanced floor of the mouth tumors who are ineligible for surgical treatment and require a non-invasive approach.

## Introduction

Tumors of the floor of the mouth represent approximately 28% of oral cavity tumors [1], and many of them are presented in advanced stages of disease at the time of diagnosis. [2] Though surgical resection is an effective and preferred modality in early stages, [3–5] treatment of advanced tumors requires a complex approach based on a combination of surgery and radiotherapy [6–9]. For patients who are considered beyond cure and surgery is not feasible, definitive radiochemotherapy is the preferred option [4, 7]. However, locoregional failure is the predominant pattern of failure in these patients [10], and the majority of local failures are identified in high-dose areas of modern radiochemotherapy due to the presence of a subpopulation of cells resistant to the standard radiation dose. Therefore, a higher total delivered radiation dose may be required [11]. However, the dose-escalation and exposure of organs at risk (OARs) to a higher dose can be associated with severe late toxicity, such as osteonecrosis [12, 13]. However, technological and technical improvements during the last years allowed for significant advances in the field of radiation therapy of head and neck cancer. The adoption of intensity-modulated radiation therapy and the use of multimodality imaging for tumor volume definition and organ at risk delineation have improved the clinical outcomes of patients with head and neck tumors [14]. Brachytherapy in combination with external beam radiotherapy (EBRT) and chemotherapy is an effective technique that leads to local dose escalation over the possibilities of up-to-date EBRT technologies [15, 16]. In technically infeasible cases, such as large tumors, patients low-performance status, and contraindications of invasive procedures, a new image-guided stereotactic radiotherapy technique is an alternative option. Recently have been published studies with stereotactic dose escalation in patients with oropharyngeal carcinomas [17, 18]. In addition, preclinical studies suggest that the effect of stereotactic radiotherapy includes the activation of ceramide apoptosis and alteration of the tumor vasculature, which may eventually help overcome tumor radioresistance [19]. Here, we present our experience with stereotactic hypofractionated radiotherapy boost combined with conventionally fractionated radiotherapy in the treatment of advanced floor of the mouth tumour.

## Materials and methods

We analyzed prospectively collected data from 37 patients treated between March 2011 and October 2018. Detailed patient characteristics are presented in Table 1. The tumor stage was defined according to the TNM classification (7th edition). All tumors were histologically confirmed as p16 negative squamous cell carcinoma. The inclusion criteria in this study were defined by the stage of disease and patients general performance status. Only patients with advanced stages of the floor of the mouth tumors were consecutively enrolled in the study. Patients with technically or medically unresectable tumors (65% technically, 32% medically, 13% both), but still enough feasible for curative intent according to multidisciplinary evaluation. The medical reasons for avoiding surgical treatment were as follows: severe malnutrition, poor performance status and multiple comorbidities (chronic kidney disease, hepatopathy, cardiac disease). All other patients were excluded from the study and were offered surgical resection (followed by chemoradiation) or palliative treatment (short course radiotherapy). The rationale for this patients selection was due to the concerns of unknown and unexpected results and toxicity of a new dose escalation regimen.

**Table 1:** Patient and tumor characteristics

Characteristic	No. of patients (%) (N = 37)
Age (median)	60 (35–82)
Gender	
Male	25 (68%)
Female	12 (32%)
Primary site	
Floor of the mouth	31 (84%)
Oral tongue	6 (16%)
ECOG performance status	
0	25 (68%)
1	12 (32%)
T stage	
2	3 (8%)
3	10 (27%)
4	24 (65%)
N stage	
0	5 (14%)
1	4 (11%)
2a	4 (11%)
2b	6 (16%)
2c	16 (43%)
3	2 (5%)
Histology	
Squamous cell carcinoma HPV negative	37 (100%)
Tumor grading	
Gr.1	25 (68%)
Gr.2	10 (27%)
Gr.3	2(5%)
SRT boost TU volume (median)	43,98cm3 (23.4–89.4)

*ECOG* Eastern cooperative oncology group

From this perspective, there was an effort to avoid harming patients with less advanced tumor stages in whom other well established and proven radical treatment modalities should be preferred. In the study, there was no control arm of patients treated with standard radical treatment modalities.

Patients were indicated for radical radiochemotherapy or hyperfractionated radiotherapy in combination with the stereotactic hypofractionated boost to the primary tumor. The radiotherapy protocol was approved by the Institutional Revision Board. Concurrent chemoradiation with cisplatin (40 mg/m^2^) administered weekly was preferred. But only a small subset of patients met the indication criteria for combined radiochemotherapy (16%). The majority of patients (84%) were unfit for chemoradiation and therefore were indicated for hyperfractionated accelerated radiotherapy (HART) [20]. Chronic kidney disease (30%), poor performance status (32%), lower BMI, elevated liver function tests and malnutrition (22%) were the main risk and exclusion factors for chemoradiation. Patients followed standard radiotherapy planning procedures. Before treatment initiation, dentition in poor condition was extracted to minimize the subsequent risk of osteoradionecrosis. None of the patients reached complete remission of the primary tumor at the end of the EBRT course. This may be related to the stage of the disease. In all patients, there was a visible tumor residue at the end of EBRT. Therefore within five days before the completion of EBRT, a new contrast-enhanced planning CT with 1 mm slice thickness was performed. After evaluation of the presence of residual tumor on CT scans and clinical examination, the stereotactic hypofractionated boost was indicated.

### Target volumes

The definition of planning target volumes in EBRT followed the recommendations of the DAHANCA, EORTC and RTOG guidelines with 3 mm isometric PTV (planning target volume) margins. Stereotactic boost (PTVboost) was defined as the gross tumor volume before EBRT with a uniform 1 mm margin.

### Dose and fractionation

**Fig. 1:**
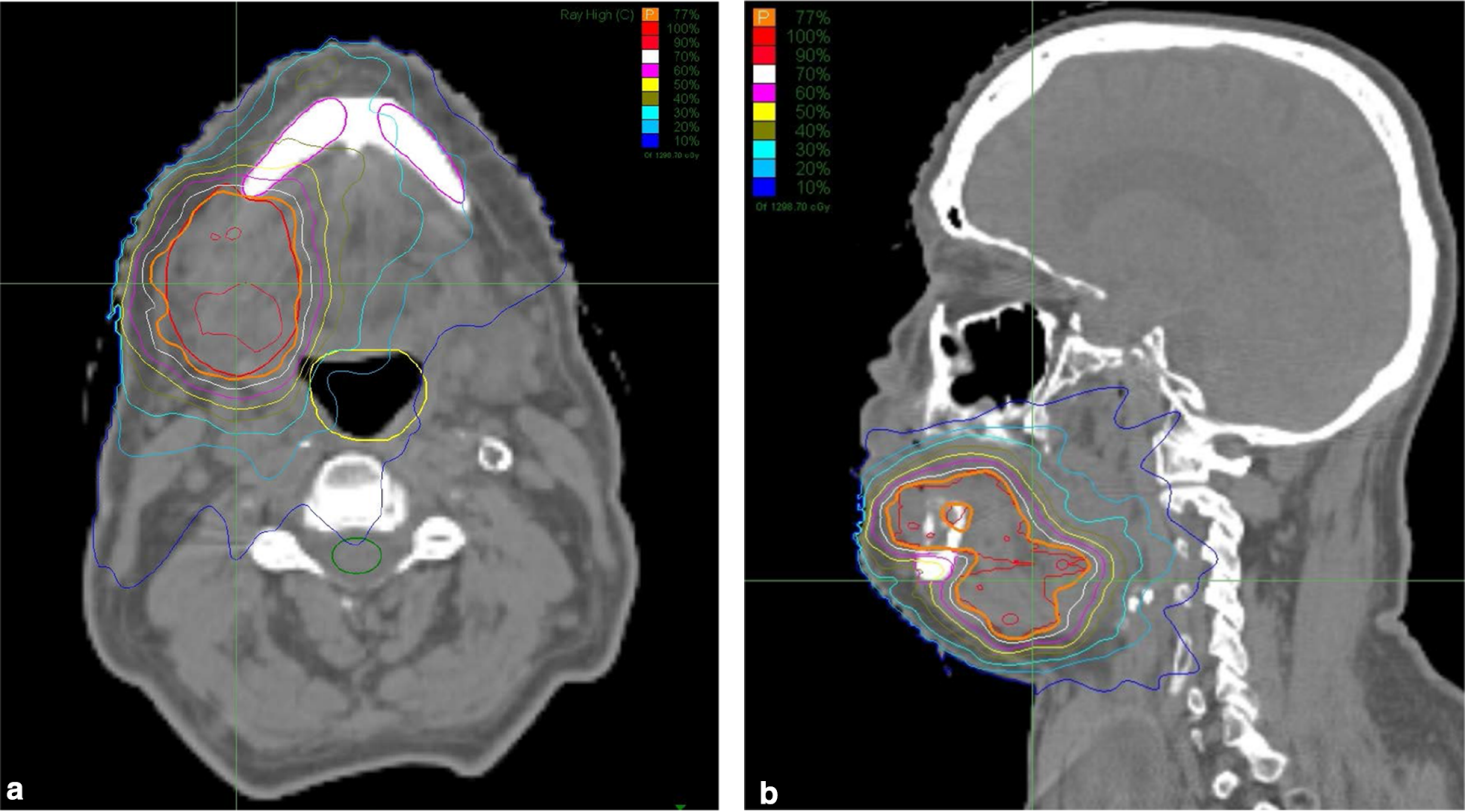
**a** Representative radiotherapy plan for the hypofractionated stereotactic boost of 10 Gy in two fractions. The persistent tumor can be seen with the gross tumor volume in (**a**) transverse and (**b**) sagital screenshots delineated by the red line. The lower jawbone, as the organ at risk, is contoured in purple. The final dose distribution takes into account tumor coverage (orange line—77% isodose) while reducing the dose to the lower jawbone

The conventional EBRT protocol was based on intensity-modulated radiation therapy (IMRT) with simultaneous integrated boost (SIB). IMRT technique was utilized to spare organs at risk. The prescribed dose to the primary tumor and bulky lymph node (LN) was 70 Gy in 35 fractions. Elective low-risk LN levels were irradiated up to 56 Gy in 35 fractions. Hyper-fractionated accelerated radiotherapy regime (HART) was used in patients ineligible for combined chemoradiation. For HART, two PTVs were defined. First PTV was determined for elective nodal level irradiation using 55 Gy in 50 fractions (i.e., 1.1 Gy/fraction twice a day at least 6 h apart). Primary tumor with bulky LN and high-risk LN levels was irradiated with 70–72.5 Gy in 50 fractions i.e., (1.4–1.45 Gy/fraction twice a day at least 6 h apart). The stereotactic hypofractionated boost continuously followed the course of conventional EBRT. Within 1 week after EBRT completion, patients received a stereotactic boost of 10 Gy in two fractions. The rationale for this fractionated regimen was supported by results from dose escalation studies in locally advanced oropharyngeal and nasopharyngeal cancer. [18, 21] The biologic effective dose for tumor effects using alpha–beta ratio of 10 (BED_10_) was defined as a sum of the total dose from EBRT (by applying the LQ model) and the dose from hypofractionated boost. BED was recalculated from the corresponding BED values by subtracting a repopulation correction factor. This factor represented a daily loss of 0,5 Gy after recalculation. The final BED corresponded to the dose of 85 Gy and 90 Gy, respectively in 2 Gy equivalent dose fractionation (EQD_10/2_). Detailed information about the radiotherapy regimes is depicted in Table 2. The median overall treatment time in the group of patients treated with radiochemotherapy was 49 days (range 49–54 days), and it was 41 days (range 36–56 days) in the group treated with HART regime. For the stereotactic boost, non-isocentric conformal beams were used. The 10 Gy dose in two fractions was prescribed to cover at least 95% of the intended irradiated volume, resulting in a prescribed isodose of 60 to 80%. During the planning process, the lower jawbone was the OAR with a high priority of minimizing the risk of osteonecrosis (Fig. 1a,b).

**Table 2:** Radiotherapy regimens and techniques

	No. of patients (%)
*EBRT regime*	
HART 72,5 Gy (1,45 Gy/fr. BID)	17 (46%)
HART 70 Gy (1,4 Gy/fr. BID)	14 (38%)
IMRT-SIB 70 Gy/56 Gy in 35fr. + cisplatin 40 mg/m2 weekly	6 (16%)
*SRT boost*	
5 Gy in 1fraction	1 (3%)
10 Gy in 2fractions	36 (97%)

*EBRT* external beam radiotherapy; *HART* hyperfractionated accelerated radiotherapy; *IMRT* intensity modulated radiotherapy; *SIB* simultaneous integrated boost; *SRT* stereotactic radiotherapy

### IGRT strategy

In EBRT, daily KV imaging and matching on bone structures was performed. In the stereotactic boost regime, online image guidance according to the bone structures (XST mode—Xsight Spine Tracking and 6D skull tracking) was used. No contrast fiducial markers were used.

### Toxicity management and scoring

The Common Terminology Criteria for Adverse Events (CTCAE) scoring system version 4 was used to evaluate acute and late radiation toxicity. Percutaneous endoscopic gastrostomy tube placement was indicated in patients with weight loss of ≥ 10% body weight before or during EBRT or the presence of abnormal swallowing and severe pain and multiple comorbidities.

### Follow-up and evaluation

The standard pre-treatment workup included the patient´s history, physical examination, CT-enhanced scans of the head and neck, chest X-ray, and abdomen ultrasound. Evaluation of the treatment response was based on physical examination and imaging (CT, PET/CT). Patients were seen every 3 months for the first year, every 4–6 months the second year, and every 6 months thereafter.

### Statistical analysis

STATISTICA 12 software was used for the statistical analysis. For the variables, tumor volume, tumor stage, and tumor grade, patients were divided into two groups, based on the median volume. The Log-Rank test was used to compare these two groups. The level of significance was 5%. Local control (LC) was defined by reaching complete remission of the primary tumor and involved lymph nodes within 12 weeks after completion of radical treatment. Progression-free survival (PFS) represented the length of time during and after the treatment without cancer progression of either local or metastatic progression. We also carried out, multivariate analysis to find relationships between local progression, metastasis, and osteonecrosis and oncological parameters, such as tumor volume, tumor stage, and tumor grading. Two sample *t* test, a univariate logistic regression model, and Pearson chi-squared test in a contingency table were used.

## Results

**Fig. 2:**
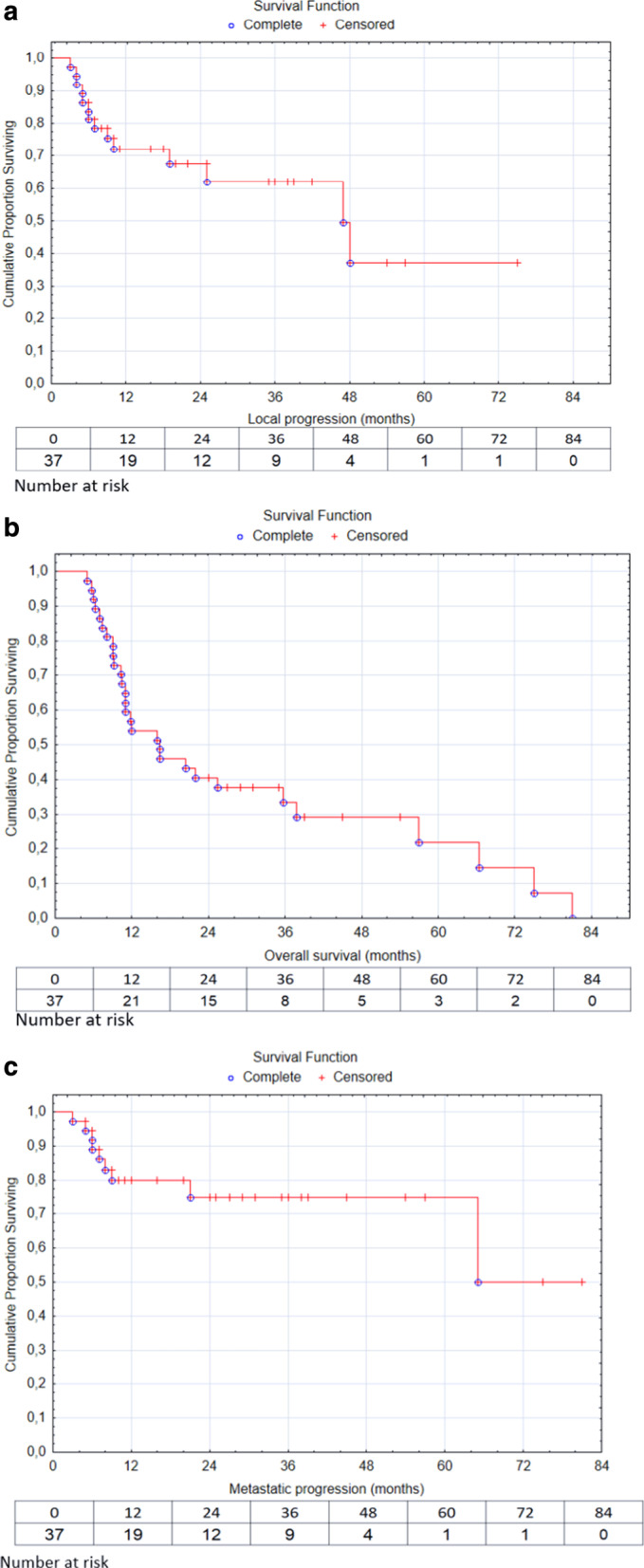
Kaplan–Meier curves for patients with oral cavity tumor (n = 37) after a median 16-month follow-up. (**a**) Local progression, (**b**) overall survival, (**c**) metastatic progression

**Fig. 3:**
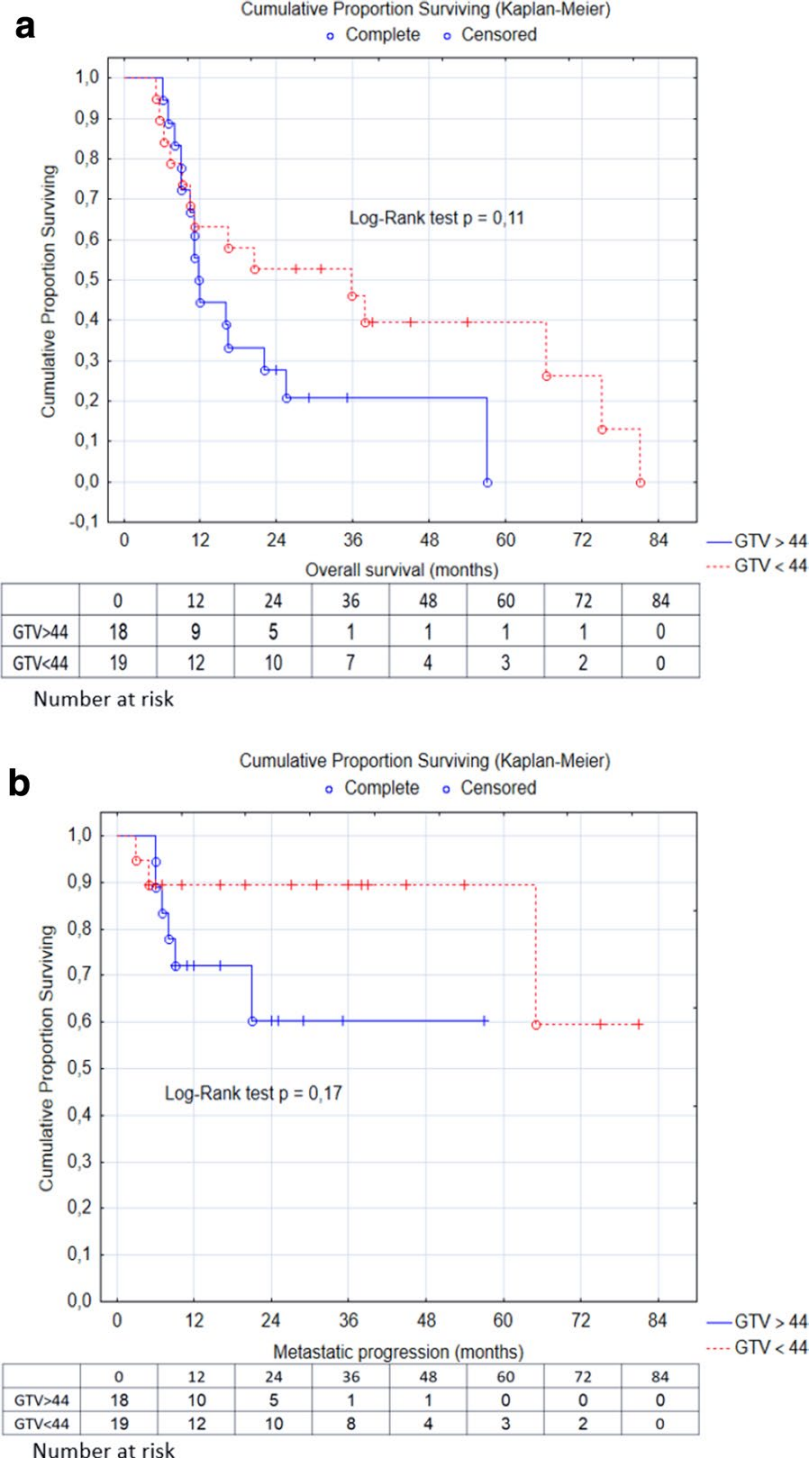
Log-Rank test showed the correlation between tumor volume and overall survival (**a**), and tumor volume and metastasis (**b**)

Thirty-six (97%) patients finished the prescribed protocol. One patient received a single-fraction stereotactic boost of 5 Gy and finished the treatment earlier due to non-compliance. Acute radiation toxicity is presented in Table 3. Dysphagia grade 2 manifested in 59% of patients. The maximum acute treatment toxicity was grade 3 mucositis and grade 3 dysphagia, both of which manifested in 10 (27%) patients who required intensification of pain treatment with opioids and nutritional support via the feeding tube. The median time to recovery from opioid dependence was 60 days (range 22–82 days). The stereotactic boost did not increase the incidence of higher acute radiation toxicity and all cases recovered within 12 weeks after completing treatment. Late radiation toxicity manifested as osteonecrosis and dysphagia. Osteonecrosis was observed in 3 (8%) patients. One of these cases was associated with local tumor progression. The two other cases were observed in patients with good local control of the primary tumor. The general management of osteonecrosis was based on a combination of surgical resection, antibiotics, and local antiseptic care. Plan parameters and dose to the lower jaw are summarized in Table 4. Twenty-six patients (70%) were not feeding tube-dependent 3 months after completion of the treatment. After a median follow-up of 16 months (range 6–82 months), local control (LC) at 2 and 5-years was 70% and 62%, respectively. Among the 37 patients, 23 (62%) showed good local control and achieved complete response and 14 (38%) experienced local progression (Fig. 2a). The median time to local progression was 6.5 months (range 3–48 months). Of the 14 patients who experienced local progression, 2 (14%) underwent salvage surgery, 1 (7%) patient was re-irradiated, and 6 (43%) were indicated for palliative chemotherapy. Five (36%) patients were ineligible for local salvage treatment or palliative chemotherapy and died due to the complications of local tumor progression. Progression-free survival (PFS) and overall survival (OS) were 57% and 49% at 2 years and 41% and 27% at 5 years, respectively. (Fig. 2b) Metastatic progression of disease was observed in 9 (24%) patients (Fig. 2c). The median time to metastasis was 7 months (range 3–47 months). Among the patients with metastatic progression, 4 (11%) had simultaneous local progression of the primary tumor. All 9 (24%) patients with metastatic progression were treated with palliative chemotherapy; 17 (46%) patients died of progression of the primary malignancy and 2 (5%) patients died of secondary malignancy. In 8 (22%) patients, the cause of death was due to other non-malignant diseases specifically due to comorbidities. Log-Rank test indicated a correlation between tumor volume and OS and metastasis. Larger tumors had a higher risk of progression and metastasis. Patients with a tumor volume ≥ 44 ccm tended to have worse OS than patients with tumor volume ≤ 44 ccm (HR = 1.96; [95% CI (0.87;4.38)]; *p* = 0.11); (Fig. 3a). A similar correlation was observed in the time to metastatic progression. Patients with tumor volume ≥ 44 ccm had a higher risk of metastasis (HR = 3.02; [95% CI (0.61; 15.08)]; *p* = 0, 17); (Fig. 3b).

**Table 3:** Acute radiation toxicity (within 90 days of radiation treatment) classified according to CTCAE version 4

Dermatitis	
Grade 1	6 (16%)
Grade 2	31 (84%)
Mucositis	
Grade 1	9 (24%)
Grade 2	18 (49%)
Grade 3	10 (27%)
Dysphagia	
Grade 1	5 (14%)
Grade 2	22 (59%)
Grade 3	10 (27%)

*CTCAE* common terminology criteria for adverse events

**Table 4:** Dose from both radiotherapy courses to the lower jaw in patients with osteonecrosis

Patients with osteonecrosis 3 (8%)	EBRT dose—D98%	SRT dose—Dmax
Case No. 1	73.8 Gy	11.,6 Gy
Case No. 2	74.4 Gy	10.,6 Gy
Case No. 3	75 Gy	10.,7 Gy

	EBRT dose—D98% (median)	SRT dose—Dmax (median)
Dose to the lower jaw of the entire study population	71.5 Gy (68.1–75)	11.6 Gy (5.6–11.68)

*EBRT* external beam radiotherapy; *SRT* stereotactic radiotherapy; *Dmax* maximum dose; *D98%* dose near maximum

## Discussion

In this study, we demonstrated the feasibility of a combination of EBRT and stereotactic hypofractionated boost in the treatment of advanced, non-resected floor of the mouth tumors in patients with other limited treatment options. Our results indicate no additional increase in acute radiation toxicity and a low rate of late toxicity. In addition, the results suggest a potential effect of dose escalation on local control. The acute radiation toxicity in the cohort was comparable to the results reported in other published studies [7, 17, 18, 22] using different radiotherapy techniques and doses. No acute CTCAE grade 4 toxicity manifested. The incidence of mucositis grade 3 (27%) and dysphagia grade 3 (27%) was associated with tumor characteristics and radiotherapy technique. However, we found no increase in the duration of acute toxicity. One patient discontinued treatment earlier due to non-compliance and a worse tolerance of radiation toxicity. In contrast, Zeno et al. [17] reported an incidence of acute grade 3 dermatitis, mucositis, and dysphagia of 15%, 15%, and 40% during treatment of T2–T4 oropharyngeal, laryngeal, and hypopharyngeal cancer by a combination of EBRT and stereotactic boost. Al-Mamgani et al. [22] reported acute radiation toxicity grade 3 dysphagia in 17–20% of patients treated for oropharyngeal cancer with a combination of EBRT and brachytherapy or stereotactic boost. In brachytherapy studies complication rate has been as high as 50–60% for acute grade 1–2 toxicities, including mucositis, dermatitis, infections, and hematoma. Moreover, the risk of mucositis grade 3 has been reported to be up to 25%, particularly when brachytherapy is combined with EBRT [23–25]. During follow-up, osteonecrosis and long-term feeding tube dependence were observed in 8% and 22% of patients. This is comparable to the results of studies with different dose-escalation techniques [12, 26, 27]. Hosni et al. [7] published the results for 108 patients with T1–T4 stage oral cavity tumors treated with definitive radiochemotherapy; after a median follow-up of 52 months, osteonecrosis developed in 6.6% of the patients. Foster et al. [28] reported the incidence of osteonecrosis and long-term feeding tube dependence in 20.7% and 10% of patients after definitive radiochemotherapy in 140 patients with oral cavity cancer. In Al-Mamgani´s study [22], the combination with brachytherapy or stereotactic boost in the treatment of oropharyngeal cancer resulted in 11% and 8% of patients having dysphagia grade ≥ 2 and feeding tube dependence in 17% and 20% of patients, respectively. In other brachytherapy studies, the incidence of late toxicity, specifically soft tissue necrosis has been reported in 10–30% of patients [13, 15, 23]. The local control rate in our cohort at 2 and 5-years was 70% and 62%, respectively. Compared to other published studies, Hosni et al. [7] recently reported the results for 108 patients with oral cavity tumors treated with definitive chemoradiotherapy. The 2-year local control rate was 80%. The 5-year local control rate was 78% for T3 stage and 72% for T4 stage. Although the results are better, in the subgroup analysis only 12% represented floor of the mouth tumors. Retrospective studies have shown that brachytherapy alone or in combination with EBRT can improve the local control rate from approximately 70% up to > 95% [15, 16, 25] in early stages. Decroix and Ghossein [29] reported outcomes in 602 patients with oral tongue tumors who underwent brachytherapy as a single-modality treatment or in combination with EBRT. In this series, the local relapse rate after treatment was 14% and 22% for T1 and T2 tumors, respectively. Pernot et al. [30] reported local control rates of 96% for T1, 85% for T2, and 64% for T3 tumors of the oral cavity treated with brachytherapy in combination with nodal neck dissection. Although brachytherapy is a very effective technique for dose escalation, it has some limitations and is mostly favoured for treatment in early stages. Yamazaki et al. [31] reported an impressive local control rate at 2- and 5-years of 89% and 71%, respectively, using a hypofractionated stereotactic CyberKnife boost in 25 patients with head and neck cancer. However, only three patients presented in the study had oral cavity tumors, which makes comparing the results with our study difficult. Evaluation of OS and PFS in a cohort of our patients with other published studies is slightly difficult due to a short median follow-up time (16 months). However, the length of follow-up is related to the number of patients in the study, the aggressiveness of the tumor and the advanced stages of the disease. From this perspective, we tried to discuss mainly 2-year rather than 5-year results. In our study, the PFS and OS were slightly worse than those reported by Foster et al. [28], who achieved 2-year OS and PFS of 73% and 61.4% and 5-year results of 63.2% and 58.7% in 140 patients with oral cavity cancer treated with definitive radiochemotherapy. A detailed analysis of the patient cohort showed that T3/T4 stage was present in 75%. Of these patients, 47.9% had oral tongue cancer and 24.3% had the floor of the mouth cancer. Tumor stage and site distribution can explain the treatment results. In contrast, Scher et al. [30] published the results for 73 patients with oral cavity tumors treated with definitive radiochemotherapy. The 2-year and 5-year OS were 24% and 15%, respectively. Disease-specific survival was 52%, 43.8%, and 38% at 1, 2, and 3 years, respectively. The disease distribution in the study was 14% stage III to 73% stage IV. However, 48% of patients had oral tongue cancer and only 19% had the floor of the mouth cancer. Hosni et al. [7] in the study of 108 patients with oral cavity cancer reached a 2-year OS of 56% and PFS of 47%, which was comparable to our results. In 5-year the OS and PFS dropped to 50% and 42%, respectively. Finally, Yamazaki et al. [31] published impressive 2-year OS and PFS of 83% and 70%, and 5-year OS of 70%, respectively. However, in the last two of these studies, the proportion of patients with the floor of the mouth tumors was only 12%, which could affect the treatment results. Statistical analysis showed that dose escalation in patients with tumor volume ≤ 44 ccm seems to be important to achieve long-term local control and survival. Patients with tumor volume > 44 ccm presented with distant failure, and dose escalation could be more controversial, though local control is still important for the quality of life. This result supports findings that larger tumors present a higher tumor mutation burden, which correlates with metastatic potential. One of the new promising options that could reduce the tumor metastatic potential is systemic maintenance therapy. A recently published study by Eder et al. [31] suggested a potential benefit of checkpoint inhibitors in the treatment of head and neck tumors with a higher tumor mutation burden. To the best of our knowledge, this study is the first to report dose escalation in patients with advanced floor of the mouth tumors. Regular follow-up and exclusion of HPV-positive tumors ensured relevant data that generate consistent output. However, our study has some limitations. Firstly a low number of patients were enrolled. In addition, only patients with advanced stages of disease and persistence of tumor after EBRT were included in the study. Also, the short follow-up time does not allow an adequate evaluation of 5-year results. The other limitations are the retrospective nature of the study and the absence of a control arm. Our study supports further investigation of stereotactic boost dose escalation in patients with the floor of the mouth cancer. We hypothesize that a stereotactic boost may improve the therapeutic index in the floor of the mouth tumors, and we are going to initiate a prospective dose-escalation phase I study.

## Conclusion

The combination of EBRT and stereotactic hypofractionated boost is a safe and effective option for dose escalation in patients with advanced floor of the mouth tumors who are ineligible for surgical treatment and require a non-invasive approach. This study demonstrated the feasibility of this combined radiotherapy regime with a moderate toxicity profile.

## Data Availability

We will provide datasets that support the results of this article on request.

## Abbreviations

HART: Hyperfractionated accelerated radiotherapy
EBRT: External beam radiotherapy
CTCAE: Common terminology criteria for adverse events
LC: Local control
GTV: Gross tumor volume
HR: Hazard ratio
CI: Confidence interval
OAR: Organs at risk
TNM: Tumor, node, metastasis
CT: Computed tomography
DAHANCA: The Danish head and neck cancer
EORTC: Europian organisation for research and treatment cancer
RTOG: Radiation therapy oncology group
PTV: Plannig target volume
IMRT: Intensity modulated radiation therapy
SIB: Simultaneous integrated boost
LN: Lymph node
IGRT: Image guided radiation therapy
XST: Xsight spine tracking
PET: Positron emission tomography
PFS: Progression free survival
HPV: Human papilloma virus
